# Diagnostic Efficacy of Xpert MTB/RIF Assay in Bronchoalveolar Lavage Fluid for Tracheobronchial Tuberculosis: A Retrospective Analysis

**DOI:** 10.3389/fmed.2021.682107

**Published:** 2021-08-18

**Authors:** Yue Sun, Qing Zhang, Qin Zhang, Chang Liu, Hong Zhang, Yinghui Fu, Yongyu Liu, Gang Hou

**Affiliations:** ^1^Institute of Respiratory Diseases, The First Hospital of China Medical University, Shenyang, China; ^2^Department of Endoscopy, Shenyang Chest Hospital, Shenyang, China; ^3^Department of Laboratory, Shenyang Chest Hospital, Shenyang, China; ^4^Institute of Respiratory Diseases, Shenyang Chest Hospital, Shenyang, China; ^5^Department of Pulmonary and Critical Care Medicine, Center of Respiratory Medicine, China-Japan Friendship Hospital, Beijing, China; ^6^Graduate School of Peking Union Medical College, Chinese Academy of Medical Sciences, Peking Union Medical College, Beijing, China; ^7^National Center for Respiratory Medicine, Beijing, China; ^8^Institute of Respiratory Medicine, Chinese Academy of Medical Sciences, Beijing, China; ^9^National Clinical Research Center for Respiratory Diseases, Beijing, China

**Keywords:** Xpert MTB/RIF, tracheobronchial tuberculosis, bronchoalveolar lavage, diagnosis, *Mycobacterium tuberculosis*

## Abstract

**Background:** The Xpert *Mycobacterium tuberculosis*/rifampin (MTB/RIF) assay has shown good diagnostic efficacy in brushing and biopsy tissue samples from patients with tracheobronchial tuberculosis (TBTB). However, its diagnostic value in bronchoalveolar lavage fluid (BALF) is still unclear. Therefore, the present retrospective study aimed to evaluate the diagnostic value of the Xpert MTB/RIF assay in BALF.

**Methods:** The clinical data of 266 patients with suspected TBTB from January 2018 to October 2020 were pooled with complete details of bronchial brush and bronchoalveolar lavage samples. Smears of the bronchial brushings were stained with Auramine O stain to detect acid-fast bacilli (AFB), and BALF samples were used for culturing MTB with the BACTEC MGIT 960 system and the Xpert MTB/RIF assay. The diagnostic performance of these methods was assessed and compared.

**Results:** A total of 266 patients suspected to have TBTB were enrolled in the final analysis. Of these patients, 179 patients were confirmed to have TBTB and 87 patients were non-TBTB. The sensitivity of the Xpert MTB/RIF assay in BALF (87.2%) was significantly higher than that of the brush smear for AFB (35.2%, *p* < 0.001). No significant difference was observed between the sensitivities of the Xpert MTB/RIF assay in BALF and MTB culture in BALF (87.2 vs. 84.9%, *p* = 0.542). The specificities of the Xpert MTB/RIF assay in BALF, MTB culture in BALF, and the bronchial brush smear were 97.7, 97.7, and 98.9%, respectively. The positive predictive value (PPV) and negative predictive value (NPV) of the Xpert MTB/RIF assay in BALF, MTB culture in BALF, and the bronchial brush smear were 98.7 and 78.7%, 98.7 and 75.9%, and 98.4 and 42.6%, respectively. Among the MTB culture-positive patients with TBTB detected by the Xpert assay, 27.0% (20/74) were identified to be resistant to RIF.

**Conclusions:** The Xpert MTB/RIF assay in BALF enables a rapid and accurate diagnosis of TBTB and identification of RIF resistance, which is crucial for timely and proper treatment. Moreover, in patients with TBTB, BALF could be used as an alternative to bronchial brushing and biopsy tissues for the Xpert MTB/RIF assay.

## Introduction

Tuberculosis (TB) is considered to be the ninth leading cause of death worldwide, and it remains one of the major global public health issues (1). Tracheobronchial tuberculosis (TBTB) is a special form of TB and is defined as *Mycobacterium tuberculosis* (MTB) infection of the tracheobronchial tree with microbial and histopathological evidence, regardless of parenchymal involvement (2). TBTB has been reported in approximately 4.1–54.3% of patients with active pulmonary TB (3, 4). Severe tracheobronchial stenosis caused by TBTB, which affects the patient's quality of life, was detected in 23.3% of patients with TBTB (5). The diagnosis of TBTB is, however, challenging because of its non-specific clinical manifestations, especially in the early stage, and unsatisfactory existing methods for acquiring microbial evidence. Thus, patients with TBTB often face a dilemma of delayed diagnosis and untimely treatment, which result in the occurrence and progression of severe tracheobronchial stenosis (6–8). Therefore, the rapid and accurate diagnosis of TBTB is crucial to minimize or prevent complications.

The Xpert MTB/rifampin (MTB/RIF) assay (Cepheid, Sunnyvale, CA, USA) is an integrated semiautomated nucleic acid amplification test designed for rapid detection of the presence of MTB and mutations associated with RIF resistance within 2 h (9). Several previous studies have proved the diagnostic value of bronchoalveolar lavage fluid (BALF) for the Xpert MTB/RIF assay in adults and children with pulmonary TB (10–12). However, because TBTB is a special type of pulmonary TB, the efficacy of its diagnosis by using different methods varies from that for pulmonary TB and even among different subtypes of TBTB because of the different characteristics of exudation, proliferation, and necrosis. Our previous study (13) showed that the sensitivities of the Xpert MTB/RIF assay of bronchial brushing and biopsy samples from TBTB patients were 57.4 and 63.9%, respectively, and the specificity for both types of samples was 100%; this proved that these results were superior to those of sputum smears, bronchial brush smears, and sputum culture. The yield of the Xpert MTB/RIF assay in bronchial brushings was significantly higher for the caseating or ulcerative subtypes than for the fibrostenotic or edematous-hyperemic subtypes (13). Bronchial biopsy and brushing samples may not be available for diagnosing bronchial fibrous stenosis and other conditions; however, in most TBTB cases, BALF is available and is a relatively less invasive and safer method. Hence, it is necessary to evaluate the diagnostic performance of the Xpert MTB/RIF assay in BALF for TBTB patients, and more specifically, to determine the diagnostic performance of the assay for different TBTB bronchoscopic subtypes. Therefore, we conducted this retrospective study to evaluate the efficacy of the Xpert MTB/RIF assay in BALF for the rapid diagnosis of TBTB.

## Materials and Methods

### Participants

This was a retrospective study conducted in the First Hospital of China Medical University and Shenyang Chest Hospital from Janurary 2018 to October 2020.

A total of 266 suspected TBTB patients were enrolled in the study through the inpatient case registration system in the hospital information system. Inclusion criteria were as follows: (1) inpatient cases suspected to have TBTB who were registered in the hospital information system and were available for follow-up after 6 months and (2) patients who underwent bronchial brush and bronchoalveolar lavage sample collection. Brushing specimens were used for smears for acid-fast bacilli (AFB), while BALF samples were used for MTB culture and the Xpert MTB/RIF assay. Exclusion criteria were as follows: (1) patients without a definite diagnosis after discharge from the hospital and after 6 months of follow-up and (2) patients with obvious pulmonary involvement instead of TBTB alone.

Definition: TBTB was confirmed by the presence of a visible tracheobronchial lesion proximal to a segmental bronchus, with histological evidence from bronchial biopsy and/or microbiological tests (sputum smear or brushing smear or BALF was positive for AFB or MTB culture was positive) (5, 14).

Clinical data of the patients were collected by two authors (YS and QinZ) from the medical databases of the hospitals. The study was approved by the Ethics Committee of China Medical University and Shenyang Chest Hospital. The ethics committee exempted obtaining informed consent from patients because of the retrospective nature of the study. Patient data were deidentified before data access and analysis.

### AFB Smear Microscopy

Three bronchial brush smears were sent to the clinical microbiology laboratory for staining directly with rapid Auramine O fluorescent stain (AO stain, Ourchem, Sinopharm Chemical Reagent Co. Ltd., Shanghai, China) for detecting AFB by microscopy (15). Sputum for AFB detection was also collected during the hospitalization. The sample was considered AFB-positive if AFB was detected in at least one bronchial brush or sputum smear microscopy.

### BALF

All patients were assessed with an Olympus electronic bronchoscopy system (1T260, Tokyo, Japan). The bronchoscopy findings revealed that TBTB showed highly variable tracheobronchial appearance, and it was classified into seven subtypes according to pathological changes reported by Chung et al. (16): actively caseating, edematous-hyperemic, fibrostenotic, tumorous, granular, ulcerative, and non-specific bronchitis type. Bronchoalveolar lavage was obtained directly from the lesion location. The total amount of 37°C saline, approximately 100 mL, was injected in equal doses, 20 mL in each dose, and finally, the lavage fluid was immediately recovered by vacuum suction (−100~-150 mmHg).

### MTB Culture and Drug Sensitivity Test

BALF specimens and sputum were processed using the BACTEC MGIT 960 rapid system (Becton Dickinson, Oxford, UK) for MTB culture (17). If the MTB culture was positive, the drug sensitivity was automatically tested through the BACTEC MGIT 960 SIRE kit (Becton Dickinson) (17).

### Xpert MTB/RIF Assay

Five milliliters of BALF specimen were taken and mixed evenly with a sample reagent buffer (SR) (containing sodium hydroxide and isopropanol) in the ratio of 2:1. The mixture was left to stand at room temperature for 15 min and then transferred to the MTB/RIF detection kit (Cepheid, USA). The reaction box was placed in the Gene Xpert instrument (Cepheid) for detection. The Xpert MTB/RIF assay has been designed to detect MTB and also mutations that confer resistance to rifampicin by using three specific primers and five unique molecular probes that target the 81-bp core region of the bacterial RNA polymerase β subunit (rpoB) gene (18–20).

### Statistical Analysis

IBM SPSS version 20.0 software (SPSS Inc., Chicago, IL, USA) was used for the statistical analysis. The sensitivity, specificity, positive predictive value (PPV), and negative predictive value (NPV) of the different diagnostic methods were calculated with two-sided 95% confidence intervals (CIs). The diagnostic performance of the different diagnostic methods for various bronchial subtypes was compared with chi-square tests. A *P*-value of <0.05 was considered to be statistically significant.

## Results

### Patient Characteristics

A total of 266 patients (155 males and 111 females) were included in the final analysis. Of these 266 patients, 179 patients were diagnosed to have TBTB. The age of the patients ranged from 13 to 91 years (mean age: 44.8 years) (Table 1). The diagnostic yield of the different diagnostic methods is shown in Table 1. The details of the remaining 87 patients with non-TBTB are also provided in Table 1. The diagnostic yield of the Xpert MTB/RIF assay in different specimens obtained from TBTB patients is shown in Supplementary Table. As Table 1 shown, 87 cases were found to be other diagnosis among people suspected of having TBTB since in the differential diagnosis.

**Table 1:** Summary of baseline patient characteristics and final diagnosis.

	**All patients**
Age (years)	44.8 ± 18.3 (median, 47; range, 13–91)
Sex (male/ female)	155/111
*Final diagnosis*	
TBTB	179
Non-TBTB	87
Lung cancer	20 (23.0%)
Non-tuberculosis mycobacteria	7 (8.0%)
Lymphoma	1 (1.1%)
Bronchitis	12 (13.8%)
Pneumonia	35 (40.2%)
AECOPD	2 (2.3%)
Bronchiectasis or hemoptysis	8 (9.2%)
Vasculitis	1 (1.1%)
Pulmonary abscess	1 (1.1%)
*Different diagnostic methods for TBTB*	
Positive MTB culture in BALF	152 (84.9%, 152/179)
Positive MTB culture in sputum	76 (82.6%, 76/92)
Positive brushing smear	63 (35.2%, 63/179)
Positive sputum smear	48 (36.9%, 48/130)

*TBTB, tracheobronchial tuberculosis; AECOPD, acute exacerbation of chronic obstructive pulmonary disease*.

Among the patients of TBTB, as for one of the initial treatment options in our patient, totally 89 patients underwent therapeutic bronchoscopy. Under bronchoscopy, if there was caseating necrosis or granular nodules or mucosal congestion and edema, cryotherapy via bronchoscope was performed; while if there was fibrous stenosis, balloon dilatation via bronchoscope was performed. None of the patients in this study received corticosteroid therapy for TBTB.

### Diagnostic Efficacy of Conventional Techniques and the Xpert MTB/RIF Assay in BALF for TBTB

The diagnostic performance of the conventional techniques and the Xpert MTB/RIF assay in BALF for TBTB is shown in Table 2. Bronchial brush smear showed AFB positivity in 35.2% (63/179, *95% CI 0.283*–*0.427*) of TBTB patients, while MTB culture in BALF specimens was positive in 84.9% (152/179, *95% CI 0.786*–*0.897*) of TBTB patients (Table 2). The sensitivity and specificity of the Xpert MTB/RIF assay in BALF to detect TBTB were 87.2% (156/179, *95% CI 0.811*–*0.915*) and 97.7% (85/87, *95% CI 0.912*−0.996), respectively (Table 2). The sensitivity and NPV of the Xpert MTB/RIF assay in BALF were significantly higher than those of bronchial brush smear microscopy (χ^2^ = *101.716, p* < *0.001;* χ^2^ = *37.141, p* < 0.001) (Table 2). No difference in sensitivity was observed between the Xpert MTB/RIF assay and MTB culture in BALF (χ^2^ = *0.372, p* = *0.542*) (Table 2).

**Table 2:** Diagnostic efficacy of the different diagnostic methods for TBTB patients.

	**Sensitivity %**	**Specificity %**	**NPV %**	**PPV %**
	**95% CI**	**95% CI**	**95% CI**	**95% CI**
Xpert MTB/RIF assay	87.2 0.811–0.915	97.7 0.912–0.996	78.7 0.696–0.858	98.7 0.950–0.998
in BALF	156/179	85/87	85/108	156/158
MTB Culture	84.9^α^	97.7	75.9	98.7
in BALF	0.786–0.897	0.912–0.996	0.667–0.833	0.949–0.998
	152/179	85/87	85/112	152/154
Brushing smear microscopy	35.2^β^ 0.283–0.427	98.9 0.929–0.999	42.6^γ^ 0.357–0.497	98.4 0.905–0.999
	63/179	86/87	86/202	63/64

*TBTB, tracheobronchial tuberculosis; CI, confidence interval; MTB, Mycobacterium tuberculosis; RIF, rifampin; BALF, bronchoalveolar lavage fluid*.

α *Compared with the Xpert MTB/RIF assay in BALF: χ^2^ = 0.372, p = 0.542*.

β *Compared with the Xpert MTB/RIF assay: χ^2^ = 101.716, p = 0.000*.

γ *Compared with the Xpert MTB/RIF assay in BALF: χ^2^ = 37.141, p = 0.000*.

### Comparison of Diagnostic Consistency of the Different Diagnostic Techniques

The diagnostic consistencies of the three different diagnostic techniques were compared (Table 3). The bronchial brush smear was not consistent with MTB culture in BALF (*Kappa coefficient* = *0.009, p* = *0.826)* and with the Xpert MTB/RIF assay in BALF *(Kappa coefficient* = *0.075, p* = *0.055*). However, the MTB culture and the Xpert MTB/RIF assay showed a fair agreement in BALF samples (*Kappa coefficient* = *0.257, p* = *0.001*). These findings revealed that the Xpert MTB/RIF assay in BALF, MTB culture in BALF, and bronchial brush smear showed a certain complementarity in their diagnostic performance for TBTB patients.

**Table 3:** Comparison of diagnostic consistency of the different diagnostic techniques.

		**Brushing smear microscopy**		**Total**
		**Positive**	**Negative**	
MTB culture	Positive	54	98	152
	Negative	9	18	27
Total		63	116	179
		**Xpert MTB/RIF**		**Total**
		**Positive**	**Negative**	
MTB culture	Positive	138	14	152
	Negative	18	9	27
Total		156	23	179
		**Brushing smear microscopy**		**Total**
		**Positive**	**Negative**	
Xpert MTB/RIF	Positive	59	97	156
	Negative	4	19	23
Total		63	116	179

*MTB, Mycobacterium tuberculosis; RIF, rifampin*.

### Diagnostic Yield of the Xpert MTB/RIF Assay According to Bronchoscopic TBTB Subtypes

The diagnostic yield of the Xpert MTB/RIF assay in different bronchoscopic TBTB subtypes were as follows: 100% (33/33) in an actively caseating subtype, 85.7% (12/14) in a granular subtype, 84% (21/25) in a fibrostenotic subtype, 89.5% (68/76) in an edematous-hyperemic subtype, and 67.9% (19/28) in a non-specific bronchitic subtype. The diagnostic performances of the Xpert MTB/RIF assay in BALF were compared for different bronchoscopic subtypes (Table 4). The tumorous and ulcerative subtypes were not included because our sample contained only three cases of these subtypes. The diagnostic rate of the Xpert MTB/RIF assay in BALF for the actively caseating subtype was significantly higher than that for the fibrostenotic subtype (χ^2^ = *5.671, p* = 0.030) (Table 4). Moreover, the diagnostic rate of the Xpert MTB/RIF assay in BALF for the non-specific bronchitic subtype was significantly lower than that for the actively caseating subtype *(*χ^2^ = *12.443, p* < *0.001*) or for the edematous-hyperemic subtype *(*χ^2^ = *6.992, p* = *0.008*) (Table 4).

**Table 4:** Diagnostic yield of the Xpert MTB/RIF in BALF according to bronchoscopic TBTB subtypes.

**SubtypesA vs. B**	**Diagnostic yield (%)**		***χ2***	***P-value***
	**Subtypes A**	**Subtypes B**		
Fibrostenotic vs. Granular	84.0 (21/25)	85.7 (12/14)	0.020	1.000^α^
Fibrostenotic vs. Caseating	84.0 (21/25)	100 (33/33)	5.671	0.030^α^
Fibrostenotic vs. Edematous-hyperemic	84 (21/25)	89.5 (68/76)	0.538	0.463
Fibrostenotic vs. Non-specific Bronchitic	84 (21/25)	67.9 (19/28)	1.859	0.173
Granular vs. Caseating	85.7 (12/14)	100 (33/33)	4.924	0.084^α^
Granular vs. Edematous-hyperemic	85.7 (12/14)	89.5 (68/76)	0.169	0.681
Granular vs. Non-specific Bronchitic	85.7 (12/14)	67.9 (19/28)	1.540	0.215
Edematous-hyperemic vs. Non-specific Bronchitic	89.5 (68/76)	67.9 (19/28)	6.992	0.008
Edematous-hyperemic vs. Caseating	89.5 (68/76)	100 (33/33)	3.749	0.103^α^
Non-specific Bronchitic vs. Caseating	67.9 (19/28)	100 (33/33)	12.443	0.000^α^
Ulcerative^β^	100 (1/1)			
Tumors^β^	100 (2/2)			

*TBTB, tracheobronchial tuberculosis; MTB, Mycobacterium tuberculosis; RIF, rifampin; BALF, bronchoalveolar lavage fluid*.

α *Fisher's exact probability method*.

β *These subtypes were excluded from all comparisons because there were only three patients*.

### RIF Resistance in TBTB Patients Based on the Xpert MTB/RIF Assay

Of the Xpert MTB/RIF-positive TBTB patients, 74 patients were also confirmed by the MGIT 960 system for drug susceptibility test. Twenty of the 74 patients with TBTB (27.0%) were found to be resistant to RIF, which was not significantly different from the result obtained for a traditional drug susceptibility test (25.7% (19/74); χ^2^ = *0.035, p* = *0.852*). The Xpert MTB/RIF assay and the traditional drug sensitivity test showed high consistency in detecting whether TBTB patients were resistant to RIF (*Kappa coefficient* = *0.965, p* < *0.001*).

## Discussion

The diagnosis and treatment of TBTB are often delayed, resulting in the development of bronchial stenosis. Therefore, a timely and accurate diagnosis of TBTB is critical. Bronchoalveolar lavage is a less invasive and technically easy method for diagnosis. In our present study, we found that the diagnostic efficacy of the Xpert MTB/RIF assay in BALF was similar to that of the MTB culture in BALF but much faster for the initiation of the treatment.

To date, sputum smear examination for AFB is considered to be a popular and rapid bacteriological examination method for diagnosing TBTB. However, it is worth noting that more than one-third of TBTB patients have negative sputum smears (21, 22), which is similar to the result of our present study (the negative smear rate was 63.1%). The bronchial brush smear also did not show satisfactory results; the detection rate was 35.2% for TBTB, similar to that reported in previous studies (23), which indicated the limited improvement in diagnosis efficacy. Although MTB culture is considered as the gold standard for diagnosing TB, it usually takes 2–8 weeks to produce results, while the Xpert MTB/RIF assay is less time-consuming (24). This retrospective study showed that the sensitivity of the Xpert MTB/RIF assay did not differ from that of MTB culture in BALF for diagnosing TBTB. The sensitivity and specificity of the GeneXpert system for diagnosing TB in an intermediate burden city were reported to be 80 and 98%, respectively (25). The PPV and NPV of the test were 92.3 and 95.1%, respectively. Therefore, the Xpert MTB/RIF assay can accurately detect MTB in BALF and prevent complications of bronchial stenosis more rapidly by enabling early proper treatment.

In our previous study (13), the Xpert MTB/RIF assay was performed on samples of bronchial brush and biopsy tissues from TBTB patients. The sensitivity of the Xpert MTB/RIF assay in BALF for TBTB patients was 87.2%, which was higher than that for bronchial brush samples (57.4%; χ^2^ = *24.822, p* = *0.000*), while the sensitivity of the Xpert MTB/RIF assay in BALF for TBTB was higher than that of the Xpert MTB/RIF assay in biopsy tissue in our previous study (63.9%, χ^2^ = *16.097, p* = *0.000*) (Supplementary Table). The comparison of the sensitivity, specificity, NPV, and PPV showed that the Xpert system had a more satisfactory “all-round” performance. The results obtained from different studies based on different diagnostic tests on different bronchoscopic samples, however, seem to be controversial. The comparison of AFB positivity showed that bronchial aspirate/bronchoalveolar lavage produced the highest diagnostic yield (51.5%) as compared to endobronchial biopsy (37.3%), transbronchial lung biopsy (45.5%), and transbronchial needle aspiration (33%) (26). For patients with pleural TB, where it is difficult to obtain sputum even after induction in some patients, bronchial lavage AFB smear, culture, and the Xpert assay were positive in 9.5, 17.9, and 26.2% patients, respectively (27). Bronchial lavage provided an immediate diagnosis in 22 (26.2%) patients (27). Bronchial lavage, although not a surrogate to pleural biopsy, offers an additional approach to the early diagnosis of pleural TB in patients unable to produce sputum. In addition to being a diagnostic tool, this method also has epidemiological significance in containing TB epidemic (27). When non-neoplastic and neoplastic bronchopulmonary lesions were pooled together, bronchial brushing showed good sensitivity (80.9%) and specificity (85.7%) as compared to bronchial washing, which had sensitivity and specificity of 47.6 and 71.4%, respectively, for the final pulmonary cytology (28). Thus, the diagnostic efficacy of the Xpert assay in different bronchoscopic samples needs to be evaluated in large-scale samples and for different TB groups. The difference in the diagnostic yield of the Xpert assay in BALF and bronchoscopic brush or biopsies might be due to the presence of necrosis or inflammatory cells that are not representative of the pathological condition. Bronchial brushing and biopsy are relatively invasive procedures, in which bleeding is a common complication. However, bronchoalveolar lavage is a procedure with a low risk of injury and bleeding to patients compared with biopsy procedure. Combined with its satisfactory “all round” performance, the Xpert assay in BALF might be a good choice for diagnosing patients suspected to have TBTB. In spite of the above advantages, what should be stressed is that among the 266 suspected TBTB patients, 87 cases were found to be other diagnosis among people suspected of having TBTB since in the differential diagnosis. Thus, differentiating from other possibilities is necessary for patients suspecting TBTB, and for this purpose, bronchial brushing and biopsy would be necessary for the confirmed diagnosis, especially when malignancies could not be excluded. In the clinical setting that TBTB is highly suspected, especially for the subtype with relatively low diagnostic yield by conventional methods, Xpert assay in BALF could be served as an alternative to bronchial brushing and biopsy.

Among the bronchoscopic subtypes, the actively caseating subtype occurs in the early stage of disease progression (16), and its diagnosis rate with the Xpert MTB/RIF assay in BALF was higher than that for the fibrostenotic subtype in our study, which is commonly considered to be the later stage of the disease (16). Previous studies have also shown that the actively caseating, edematous-hyperemic, and fibrostenotic subtypes have different degrees of bronchial lumen stenosis (16) and the worst prognosis (29). Our study showed that the diagnostic rate of the Xpert MTB/RIF assay in BALF for the actively caseating or edematous-hyperemic subtype was higher than that for the non-specific bronchitic subtype; this finding indicating the potential influence of different specific characteristics of different bronchoscopic subtypes on the diagnostic yield of Xpert MTB/RIF assay in BALF. For the earlier and more active stage, the diagnosis rate would be higher, while for the later and more fibrostenotic state, the diagnosis rate might be lower. And our finding further demonstrates the superior diagnostic value of the Xpert MTB/RIF assay in BALF for TBTB patients with bronchial lumen stenosis. Another merit of the Xpert MTB/RIF assay is that it could rapidly detect whether TBTB patients were resistant to RIF. Consistent with related reports, the Xpert MTB/RIF assay showed a high agreement with traditional drug sensitivity tests in our study (30–32).

The present study has several limitations. First, this was a retrospective study based on electronic medical records as the source of patient data. Therefore, more prospective studies are needed. Second, most TBTB cases were concomitant TBTB in pulmonary TB patients. However, patients with obvious pulmonary involvement instead of TBTB alone were excluded in order to evaluate the diagnostic efficacy of Xpert MTB/RIF assay in BALF. Thus, the sample size was small, especially for the special subtypes of ulcerative and tumor. The corresponding results of TBTB patients with tumor subtype and ulcer subtype were not analyzed due to the extremely small sample size. Third, the sensitivity of the Xpert MTB/RIF assay in BALF, brushing tissues, and biopsy tissues from TBTB patients could not be directly compared.

In conclusion, for patients suspected to have TBTB, the Xpert MTB/RIF assay in BALF is a more sensitive method to detect MTB than the conventional methods of bronchial brush smear. BALF could be considered as an alternative to biopsy tissue and brushings for the Xpert MTB/RIF assay for TBTB patients. The Xpert MTB/RIF assay in BALF showed a satisfactory “all round” performance, which could enable an early appropriate treatment.

## Data Availability Statement

The original contributions generated for this study are included in the article/Supplementary Material, further inquiries can be directed to the corresponding author/s.

## Ethics Statement

The study was approved by the Ethics Committee of China Medical University and Shenyang Chest Hospital. The ethics committee exempted obtaining informed consent from patients because of the retrospective nature of the study.

## Author Contributions

GH conceived the study. GH and YL designed the study. QingZ and CL conducted bronchial brush and bronchoalveolar lavage. YS and QingZ wrote the manuscript. GH, YL, QingZ, CL, YS, QinZ, HZ, and YF contributed to data collection and data analysis. All authors read and approved the final manuscript.

## Conflict of Interest

The authors declare that the research was conducted in the absence of any commercial or financial relationships that could be construed as a potential conflict of interest.

## Publisher's Note

All claims expressed in this article are solely those of the authors and do not necessarily represent those of their affiliated organizations, or those of the publisher, the editors and the reviewers. Any product that may be evaluated in this article, or claim that may be made by its manufacturer, is not guaranteed or endorsed by the publisher.
