# Supplementary material for: Diagnostic Efficacy of Xpert MTB/RIF Assay in Bronchoalveolar Lavage Fluid for Tracheobronchial Tuberculosis: A Retrospective Analysis
Source: Front Med (Lausanne). 2021 Aug 18;8:682107. doi: 10.3389/fmed.2021.682107 (PMC8416264; doi:10.3389/fmed.2021.682107)
Supplement: Supplementary file 1 [file Table_1.docx]

**Supplementary Table** Diagnostic yield of the Xpert MTB/RIF in different specimens obtained from patients with TBTB

| Specimens  A vs B | Diagnostic yield (%) | | *χ2* | *P-value* |
| --- | --- | --- | --- | --- |
|  | Specimen A | Specimen B |  |  |
| BALF vs Bronchial brushings | 87.2  (156/179) | 57.4  (35/61) | 24.822 | 0.000 |
| BALF vs Biopsy | 87.2  (156/179) | 63.9  (39/61) | 16.097 | 0.000 |

TBTB, tracheobronchial tuberculosis; MTB, mycobacterium tuberculosis; RIF, rifampin; BALF, bronchoalveolar lavage fluid.
